# Expression of deleted in liver cancer 1 and plasminogen activator inhibitor 1 protein in ovarian carcinoma and their clinical significance

**DOI:** 10.1186/1756-9966-32-60

**Published:** 2013-08-30

**Authors:** Fang Ren, Huirong Shi, Gong Zhang, Ruitao Zhang

**Affiliations:** 1 Department of Obstetrics and Gynecology, the First Affiliated Hospital of Zhengzhou University, NO.1 Jian She Road, Zhengzhou 450052, China; 2 Department of Hepatobiliary and Pancreatic Surgery, the First Affiliated Hospital of Zhengzhou University, NO.1 Jian She Road, Zhengzhou 450052, China

**Keywords:** Ovarian cancer, Deleted in liver cancer 1, Plasminogen activator inhibitor 1, Invasion, Metastasis, Prognosis

## Abstract

**Background:**

The deleted in liver cancer 1 (DLC1) and plasminogen activator inhibitor 1 (PAI-1) are known to be closely associated with tumor growth and metastasis in several kinds of human tumors. The aim of this study was to investigate the expression of DLC1 and PAI-1 in ovarian carcinoma, and evaluate their relations with the prognosis of ovarian carcinoma.

**Methods:**

Immunohistochemical staining and Western blot were used to examine the expressions of DLC1 and PAI-1 protein in 25 specimens normal ovarian tissues, 52 specimens of serous cystadenocarcinoma tissues and 23 specimens of mucinous cystadenocarcinoma tissues. Chi-square test, Logistic regression and Partial Correlate analysis were performed to evaluate the association between DLC1 and PAI-1 with clinicopathological characteristics. Overall survival was estimated by Kaplan-Meier curves and multivariate Cox analysis. The relationships between DLC1 and PAI-1 protein expression were analyzed by Pearson’s correlation coefficient.

**Results:**

The expression of DLC1 protein in ovarian carcinoma tissues was significantly lower than that in normal ovarian tissues, but it was converse for PAI-1. In ovarian carcinoma, the expression of DLC1 was significantly associated with advanced FIGO stage, ascites and positive lymph node metastasis, whereas PAI-1 protein was closely related with advanced FIGO stage, poor histological differentiation and lymph node metastasis. The expression of DLC1 was negatively correlated with PAI-1 in ovarian carcinoma. Ovarian cancer patients with negative expression of DLC1 and positive expression of PAI-1 had the worst overall survival time compared to other patients.

**Conclusions:**

The expression of DLC1 and PAI-1 were closely related with the metastasis and invasion of ovarian carcinoma, only the combination of DLC1 and PAI-1 could serve as an independent prognostic factor of ovarian carcinoma.

## Background

Epithelial ovarian cancer (EOC) is the leading cause of cancer-related death in women diagnosed with gynecologic malignancies [1]. The reported 5-year survival rates for women with EOC are only 25% ~ 30%, in spite of recent advances in cytoreductive surgery and chemotherapeutic agents. Recent advances in histopathology and cytogenetics have provided insights into pathophysiologic features and natural history of EOC [2,3].

Deleted in liver cancer 1 (DLC1), a GTPase-activating protein (GAP) domain containing tumor suppressor which localizes at focal adhesions, is considered as a potential tumor suppressor for many malignant tumors [4]. DLC1 is a focal adhesion molecule that plays a role in preventing cell transformation in the liver as well as many other tissues [5,6]. Using gene transfection technology, DLC-1 gene high expression can inhibit the ovarian cancer cell line OVCAR-3 cells proliferation significantly [7]. Plasminogen activator inhibitor 1 (PAI-1) is the key inhibitor of the plasminogen activation system (PAS), PAI-1 participate in the pathophysiology of a number of diseases such as atherosclerosis, restenosis, and cancer [8-11]. The canonical view of PAI-1 as an inhibitor of tPA and uPA cannot fully account for a mechanism whereby PAI-1 contributes to the disease. As an effective specific uPA inhibitor, PAI-1 participated in the formation of uPA/uPAR/PAI-1 complexes, the regulation of uPA activity and the position of uPA receptor on the cell membrane. By this means, PAI-1 maintained the balance of the extracellular protein degradation and prevented the excessive degradation of ECM, thus inhibited tumor metastasis [12,13]. In ovarian cancer cell lines, cell migratory and invasive phenotype is reduced by active PAI-1 due to its ability to inhibit plasminogen activation compared to their plasminogen activator system profiles [14].

In normal prostate epithelial cells, silencing of DLC1 by RNAi can upregulate PAI-1 expression and reduce cell migration [15]. However, the relations between the expression of DLC1 and PAI-1 and invasion, metastasis and prognosis of epithelial ovarian carcinoma were still unknown. In this study, we detected the expression of DLC1 and PAI-1 in ovarian carcinoma, evaluated the associations between their expressions and clinical pathologic factors, and explored the role of DLC1 and PAI-1 in the prognosis of epithelial ovarian carcinoma.

## Material and methods

### Patients and tissue samples

100 ovarian specimens were obtained from the patients during surgeries in the Department of gynaecology and obstetrics, the First Affiliated Hospital of Zhengzhou University (from January 2007 to October 2010), which consisted of 25 specimens normal ovarian tissues (obtained from patients who underwent hysterectomy and oophorectomy for multiple uterinemyoma other than ovarian tumors), 75 specimens of ovarian carcinoma tissues (contains 52 serous cystadenocarcinoma and 23 mucinous cystadenocarcinoma). Every I-II stage EOC patient underwent satisfy cytoreductive surgery, every III-IV stage EOC patient underwent unsatisfy cytoreductive surgery. The tissue samples were collected after surgery resection immediately and saved in liquid nitrogen promptly. The median age of all the patients was 52 years old (range 19 to 73). All patients did not receive preoperative radiochemotherapy. The median follow-up was 36 months (range 9 to 70 months), 48 patients were still survival at the end of follow-up. The tissue sample collection all obtained the consent of enrolled patients before the operation, and the present study was approved by the local Ethics Committee of Zhengzhou University. The collecting of tissue samples was supervised by a pathologist, and all the tissue samples were verified by two pathologists before IHC independently by HE stain.

### Immunohistochemistry

The antibodies used in the Immunohistochemistry, following manufacturer’s protocols, were anti-DLC1 and anti- PAI1 (Santa Cruz, USA). Immunohistochemistry staining used DAKO EnVision System (Dako Diagnostics, Switzerland) following the protocol. For DLC1 and PAI-1 protein, staining localized in the cytoplasm was considered positive. The immunoreactive score was calculated followed Remmele’s method [16]. The percentage of positive cells was scored as follows: without stain scored - (0), less than 10% positive cells scored + (1), 10–50% ++ (2), 51–80% +++ (3) and more than 80% ++++ (4). The staining intensity was also scored on a four-tiered scale (negative scored 0, low intensity positive staining 1, moderate intensity positive staining 2, and strong intensity positive staining 3). The staining intensity score plus the positive cell score is the overall score. 0 score was negative staining (−), more than 2 scores were positive staining (+), more than 6 scores was strong positive (++). Immunoreactive score was performed by two pathologists independently.

### Western blotting

The antibodies used in the Western blot, following manufacturer’s protocols, were anti-DLC1, anti-PAI-1 and anti-β-actin (Santa Cruz, USA). Tissue lysates containing equal amounts of total protein were separated by SDS-PAGE. To detect proteins of interest, enhanced chemiluminescence system was used according to the supplier’s protocol (Lumi-Light Western Blotting substrate; Roche). Relative levels of proteins were estimated densitometrically using β-actin as internal reference.

### Statistical analysis

SPSS 17.0 software was used for the statistical analysis. Continuous variables were expressed as $\bar{X}\pm s$. Chi-square test, Logistic regression analysis and Partial Correlate were performed to evaluate the association between DLC1 and PAI-1 with clinicopathological characteristics. Overall survival was estimated by Kaplan-Meier curves and multivariate Cox analysis. The relationships between DLC1 and PAI-1 protein expression were analyzed by Pearson’s correlation coefficient. Results were considered statistically significant when *P* less than 0.05.

## Results

### Expression of DLC1 and PAI-1 in epithelial ovarian cancer tissues and normal ovarian tissues

**Figure 1:**
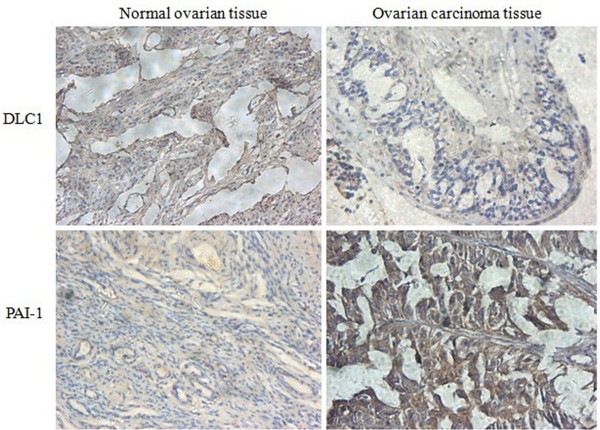
**Positive expression of DLC1 and PAI-1 in different ovarian tissues detected by immunohistochemistry staining.** Normal ovary cells showed a higher staining of DLC1 (Up-left), but ovarian cancer cells showed lower density staining (Up-right); normal ovary cells showed a lower staining of PAI-1 (Down-left), but ovarian cancer cells showed higher density staining (Down-left). Immunoreactive Score method performed followed Remmele’s method, the number of positive-staining cells in 10 representative microscopic fields was counted, and the percentage of positive cells was calculated (DAB staining, ×400).

**Figure 2:**
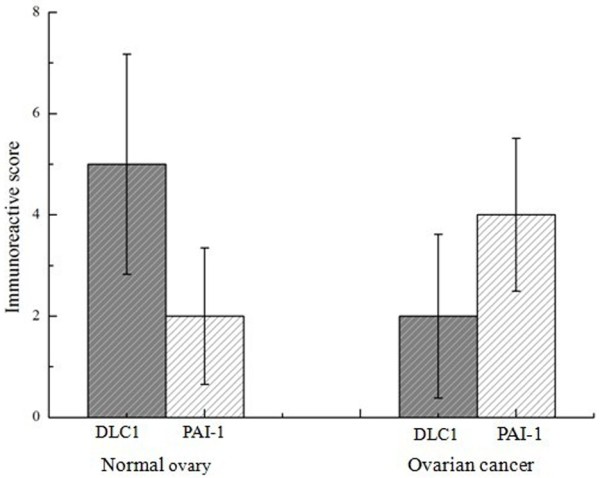
**The immunoreactive scores of DLC1 and PAI-1 in different ovarian tissues detected by immunohistochemistry staining.** Normal ovary showed a higher score of DLC1, but ovarian cancer showed lower score, significant differences were observed (*P* < 0.05); normal ovary showed a lower score of PAI-1, but ovarian cancer showed higher score, significant differences were observed (*P* < 0.05). Bar graphs show the positive score of DLC1 and PAI-1 protein.

**Figure 3:**
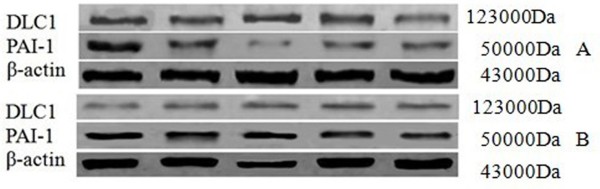
**Expression of DLC1 and PAI-1 in normal ovarian tissue (A) and ovarian cancer tissues (B) detected by Western Blotting.** Interest bands were presented by Western Blotting from different tissue samples, each protein band represents one random specimen tissue. Normal ovary showed a higher expression of DLC1, but ovarian cancer showed lower expression; normal ovary showed a lower expression of PAI-1, but ovarian cancer showed higher expression.

**Figure 4:**
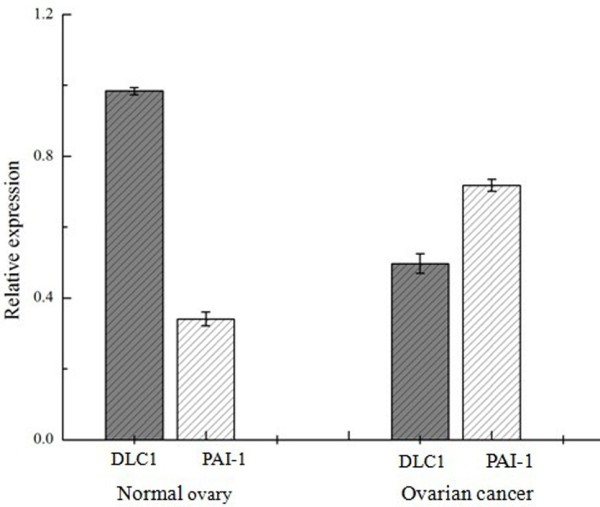
**Bar graph of the Western Blotting assay.** Each bar represents the relative value of DLC1 and PAI-1 protein**,** significant differences were observed between normal ovary and ovarian carcinoma (*P* < 0.05).

Positive staining for DLC1 observed in malignant and normal ovarian tissues were 33/75 (44.0%) and 25/25 (100.0%) respectively, but were 51/75 (68.0%) and 9/25 (36%) for PAI-1 (Figures 1 and 2). The Western Blotting showed that the expression of DLC1 protein in normal and malignant ovarian tissues were (0.984 ± 0.010) and (0.497 ± 0.028), but (0.341 ± 0.019) and (0.718 ± 0.017) for PAI-1 (Figures 3 and 4). The expression of DLC1 in ovarian carcinoma tissues was significantly lower than that in normal ovarian tissues (*P* < 0.05), whereas it was converse for PAI-1.

### Association of DLC1 and PAI-1 expression with the clinicopathologic characteristics of ovarian cancer

As shown in Table 1, the expression of DLC1 and PAI-1 were significantly associated with FIGO stage and lymph node metastasis in ovarian carcinoma. In addition, DLC1 was also related with ascites, and PAI-1 was related with histological differentiation.

**Table 1:** Relations between expression of DLC1 and PAI-1 in ovarian cancer and clinical characteristics of epithelial ovarian cancer

**Group**	**n**	**DLC1**		***χ***^**2**^	***P***	**PAI-1**		***χ***^**2**^	***P***
		**+**	**%**			**+**	**%**		
Age									
<50	27	11	40.7	0.182	0.670	20	74.1	0.715	0.398
≥50	48	22	45.8			31	64.6		
Histological type									
Serous	52	21	40.4	0.900	0.343	35	67.3	0.037	0.847
Mucinous	23	12	52.2			16	69.6		
FIGO stage									
I ~ II	32	19	59.4	5.355	0.021^*^	16	50.0	8.311	0.004^*^
III ~ IV	43	14	32.6			35	81.4		
Histological differentiation									
G1	16	9	56.3	5.372	0.068	7	43.8	6.359	0.042^*^
G2	25	14	56.0			17	68.0		
G3	34	10	29.4			27	79.4		
Lymph metastasis									
YES	33	9	27.3	6.692	0.010^*^	28	84.8	7.688	0.006^*^
NO	42	24	57.1			23	54.8		
Ascites									
YES	52	17	32.7	8.799	0.003^*^	37	71.2	0.775	0.379
NO	23	16	69.6			14	60.9		

^*^Chi-square test. Compared with normal ovarian tissues *P* < 0.05.

### The correlation between DLC1 and PAI-1 in epithelial ovarian carcinoma

Among the 75 specimens of EOC, there were 15 positive for DLC1 and negative for PAI-1, as well as 33 negative for DLC1 and positive for PAI-1. This result suggests a negative correlation between the expression of DLC1 and PAI-1 (*r* = −0.256, *P* = 0.027).

### Associations of DLC1 and PAI-1 expression with the prognosis of ovarian cancer

**Figure 5:**
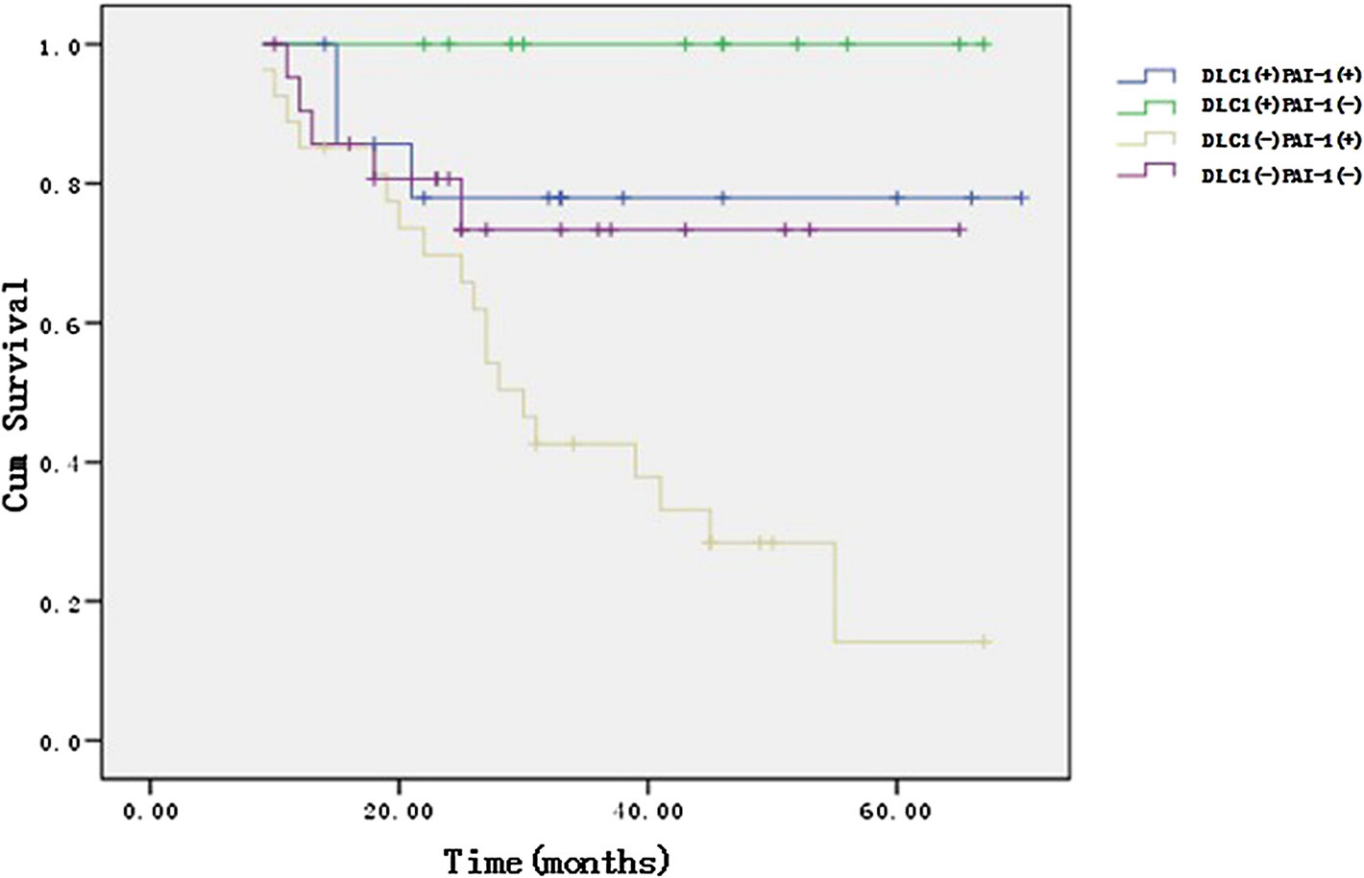
**Survival curves showing the association between overall survival and combining DLC1 and PAI-1 expression.** Ovarian cancer patients with negative expression of DLC1 and positive expression of PAI-1 had the worst overall survival time compared to other patients.

Partial Correlate analysis showed the expression of DLC1 was negatively related with FIGO stage (*P* = 0.015), ascites (*P* = 0.043), lymph node metastasis (*P* = 0.021), but positively related with prognosis (*P* = 0.009). The expression of PAI-1 was positively related with FIGO stage (*P* = 0.011), histological differentiation (*P* = 0.036), lymph node metastasis (*P* = 0.027), but negatively related with prognosis (*P* = 0.018). Logistic Regression analysis indicated the expression of DLC1 was closely related with FIGO stage (*P* = 0.032), the expression of PAI-1 was closely related with lymph node metastasis (*P* = 0.048), and the expression of DLC1 combined with PAI-1 were significant correlative factors with prognosis (*P* < 0.05). Furthermore, Kaplan-Meier survival curves demonstrated that ovarian cancer patients with negative expression of DLC1 and positive expression of PAI-1 had the worst overall survival time compared to other patients (Figure 5). Multivariate Cox analysis showed that only DLC1 combined with PAI-1 expression (*P* < 0.05) were independent risk factors of prognosis.

## Discussion

Invasion and metastasis are characteristics of malignant solid tumors, and many mechanisms are involved in these processes. Advanced FIGO stage, ascites and positive lymph node metastasis are the critical factors in the invasion and metastatic spread of ovarian cancer [3,17,18]. Furthermore, they are related with prognosis in patients with ovarian cancer. However, the mechanism of the invasion and metastasis events in ovarian cancer has yet to be defined.

DLC1 was expressed in many normal tissues, but its expression was lost or down regulated in various cancers including liver, breast, lung, brain, stomach, colon and prostate cancers, which suggested that DLC1 may function as a tumor suppressor [6,19-22]. Re-expression of DLC1 in liver, breast, lung cancer cell lines inhibits cancer cell growth [23]. Likewise, reintroduction of DLC1 breast cancer cell lines results decreased tumorigenic growth, supporting its major role as a tumor suppressor [24,25]. However, tumor malignant transformation and progression to metastasis are often associated with changes in cell cytoskeletal organization and cell-cell adhesion. DLC1 gene can encode a RhoGAP protein that inactivates Rho GTPases, which are critically involved in the regulation of cytoskeleton and cell migration [4,26]. Recently, abnormal, low, or lack of DLC1 expression was found to be associated with the metastasis of breast and hepatocellular cancers, suggesting that DLC1 plays an important role not only in tumorigenesis but also in metastasis [5,27]. The gene expression profiles of metastatic and non-metastatic sublines of the parental MDA-MB-435 breast cancer cell line were compared and DLC1 was down-expressed in the metastatic subline. Restoration of DLC1 in metastatic cell line leads to the inhibition of migration and invasion in cell culture assays and a significant reduction in metastases in nude mouse experiments [27]. In present study, although expression of DLC1 was observed in ovarian carcinoma tissues, it was significantly lower than that in normal ovarian tissues. The expression of DLC1 was significantly associated with advanced FIGO stage, ascites, and positive lymph node metastasis, which suggested that DLC1 might be involved in the invasion and metastasis of ovarian cancer.

Plasminogen activator inhibitor-1 (PAI-1) belongs to the serine protease inhibitor superfamily, previous studies about PAI-1 mainly focused on the inhibition of fibrinolysis [7,13,28,29]. Recently, it has been found that PAI-1 is involved in the pathophysiological process about degradation of extracellular matrix, cell migration, metastasis and various reactions of cellular signal transduction [8,30,31]. In a retrospective study, a strong association between elevated levels of PAI-1 and aggressive disease recurrence has been found [32]. Elevated expression of PAI-1 protein was associated with increased risk of distant metastasis in renal cancer [33,34]. High PAI-1 expression levels were associated with malignancy and PAI-1 is a strong predictor of local, as well as distant metastasis [35]. The positive rates of PAI-1 was significantly higher in epithelial ovarian cancer than in benign ovarian tumor which was detected by immunohistochemistry, and PAI-1 was an independent factor for overall survival [36]. PAI-1 was significantly overexpressed in the tumor epithelium of ovarian cancer in comparison to the ovarian epithelium of benign ovarian tumor and normal ovary, which was detected by immunohistochemistry and ELISA [37]. These studies suggested that PAI-1 might play an important role in the invasion and metastasis of solid tumors. In this study, western blot and immunohistochemistry analysis showed high PAI-1 protein levels in ovarian carcinoma tissues, which was significantly higher than that in normal ovarian tissues. We also found that the expression of PAI-1 protein were significantly associated with advanced FIGO stage, poor histological differentiation and lymph node metastasis, suggesting that PAI-1 was implicated in the invasion and metastasis of ovarian cancer.

However, the interaction mechanisms of DLC1 and PAI-1 that involve in the invasion and metastasis in tumor cells had not been well studied. Recently, in normal prostate epithelial cells DLC1 modulates the expression of PAI-1, which is a negative regulator for cell migration, in a GAP domain and EGFR-MEK-dependent manner was demonstrated [15]. While, independent of PAI-1, the interaction of DLC1 with tensin members positively regulates cell migration. In our study, the expression of DLC1 and PAI-1 in ovarian carcinoma tissues showed an obvious negative correlation, which indicated DLC1 and PAI-1 might be closely related to the tumorigenesis of ovarian carcinoma, and linked in the progress of tumor invasion and metastasis.

In Partial Correlate analysis, the expression of DLC1 and PAI-1 were closely related with the metastasis and invasion of ovarian carcinoma, both DLC1 and PAI-1 could be used to assess the prognosis respectively, but only the combination of DLC1 and PAI-1 was an independent prognostic factor of ovarian carcinoma which was confirmed by Logistic Regression analysis. As the survival analysis data shown in Figure 5, patients with low expression of DLC1 or high expression of PAI-1 both had reduced survival time, especially when DLC1 was low expression and PAI-1 was high expression at the same time. Those results strengthened the notion that combination of DLC1 and PAI-1 could serve as an independent prognostic factor of ovarian carcinoma.

## Conclusions

The enrolled samples were limited, and the follow-up time was varying, but this study presented some valuable results. Upon the present results, the expression of DLC1 and PAI-1 were closely related with the metastasis and invasion of ovarian carcinoma, both DLC1 and PAI-1could be used to assess the prognosis respectively, but only the combination of DLC1 and PAI-1 could serve as an independent prognostic factor of ovarian carcinoma. In next steps, the potential signaling pathways that regulate DLC1 and PAI-1 expression in ovarian cancer cell migration and invasion will be discussed.

## Abbreviations

DLC1: Deleted in liver cancer 1; PAI-1: Plasminogen activator inhibitor 1; FIGO: International Federation of Gynecology and Obstetrics; EOC: Epithelial ovarian cancer; GAP: GTPase-activating protein; PAS: Plasminogen activation system; EGFR: Epidermal growth factor receptor; MEK: Mitogen-activated protein kinase.

## Competing interests

The authors declare that they have no competing interests.

## Authors’ contributions

RF participated in design of the study, carried out molecular studies, drafted manuscript and performed statistical analysis. SH participated in design of the study and reviewed manuscript. ZG and ZR carried out immunohistochemistry and western blotting analysis. All authors read and approved the final manuscript.

## References

[B1] Roett MA Evans P Ovarian cancer: an overview Am Fam Physician 2009 80 609 616 19817326

[B2] Kim A Ueda Y Naka T Enomoto T Therapeutic strategies in epithelial ovarian cancer J Exp Clin Cancer Res 2012 13 31 14 10.1186/1756-9966-31-14 PMC3309949 22330607

[B3] Chen SS Michael A Butler-Manuel SA Advances in the treatment of ovarian cancer: a potential role of antiinflammatory phytochemicals Discov Med 2012 13 7 17 22284780

[B4] Kim TY Vigil D Der CJ Juliano RL Role of DLC-1, a tumor suppressor protein with RhoGAP activity, in regulation of the cytoskeleton and cell motility Cancer Metastasis Rev 2009 28 77 83 10.1007/s10555-008-9167-2 19221866 PMC2757774

[B5] Liao YC Lo SH Deleted in liver cancer-1 (DLC-1): a tumor suppressor not just for liver Int J Biochem Cell Biol 2008 40 843 847 10.1016/j.biocel.2007.04.008 17521951 PMC2323245

[B6] Kim TY Lee JW Kim HP Jong HS Kim TY Jung M Bang YJ DLC-1, a GTPase-activating protein for Rho, is associated with cell proliferation, morphology, and migration in human hepatocellular carcinoma Biochem Biophys Res Commun 2007 355 72 77 10.1016/j.bbrc.2007.01.121 17292327

[B7] Liu H Shi H Hao Y Zhao G Yang X Wang Y Li M Liu M Effect of FAK, DLC-1 gene expression on OVCAR-3 proliferation Mol Biol Rep 2012 39 10665 10670 10.1007/s11033-012-1956-6 23079702

[B8] Cesari M Pahor M Incalzi RA Plasminogen activator inhibitor-1 (PAI-1): a key factor linking fibrinolysis and age-related subclinical and clinical conditions Cardiovasc Ther 2010 28 e72 e91 10.1111/j.1755-5922.2010.00171.x 20626406 PMC2958211

[B9] Gramling MW Church FC Plasminogen activator inhibitor-1 is an aggregate response factor with pleiotropic effects on cell signaling in vascular disease and the tumor microenvironment Thromb Res 2010 125 377 381 10.1016/j.thromres.2009.11.034 20079523 PMC2860057

[B10] Samarakoon R Goppelt-Struebe M Higgins PJ Linking cell structure to gene regulation: signaling events and expression controls on the model genes PAI-1 and CTGF Cell Signal 2010 22 1413 1419 10.1016/j.cellsig.2010.03.020 20363319 PMC2903658

[B11] Arteel GE New role of plasminogen activator inhibitor-1 in alcohol-induced liver injury J Gastroenterol Hepatol 2008 23 Suppl 1 S54 S59 18336665 10.1111/j.1440-1746.2007.05285.x PMC2413330

[B12] Czekay RP Loskutoff DJ Unexpected role of plasminogen activator inhibitor 1 in cell adhesion and detachment Exp Biol Med (Maywood) 2004 229 1090 1096 15564434 10.1177/153537020422901102

[B13] Cho SH Ryu CH Oh CK Plasminogen activator inhibitor-1 in the pathogenesis of asthma Exp Biol Med (Maywood) 2004 229 138 146 14734792 10.1177/153537020422900202

[B14] Whitley BR Palmieri D Twerdi CD Church FC Expression of active plasminogen activator inhibitor-1 reduces cell migration and invasion in breast and gynecological cancer cells Exp Cell Res 2004 296 151 162 10.1016/j.yexcr.2004.02.022 15149846

[B15] Shih YP Takada Y Lo SH Silencing of DLC1 upregulates PAI-1 expression and reduces migration in normal prostate cells Mol Cancer Res 2012 10 34 39 10.1158/1541-7786.MCR-11-0450 22064653 PMC3262057

[B16] Remmele W Stegner HE Recommendation for uniform definition of an immunoreactive score (IRS) for immunohistochemical estrogen receptor detection (ER-ICA) in breast cancer tissue Pathologe 1987 8 138 140 3303008

[B17] Dutta S Wang FQ Phalen A Fishman DA Biomarkers for ovarian cancer detection and therapy Cancer Biol Ther 2010 9 668 677 10.4161/cbt.9.9.11610 20372062

[B18] Matsuo K Sheridan TB Yoshino K Miyake T Hew KE Im DD Rosenshein NB Mabuchi S Enomoto T Kimura T Sood AK Roman LD Significance of lymphovascular space invasion in epithelial ovarian cancer Cancer Med 2012 1 156 164 10.1002/cam4.31 23342265 PMC3544453

[B19] Durkin ME Yuan BZ Thorgeirsson SS Popescu NC Gene structure, tissue expression, and linkage mapping of the mouse DLC-1 gene (Arhgap7) Gene 2002 288 119 127 10.1016/S0378-1119(02)00462-6 12034501

[B20] Guan M Zhou X Soulitzis N Spandidos DA Popescu NC Aberrant methylation and deacetylation of deleted in liver cancer-1 gene in prostate cancer: potential clinical applications Clin Cancer Res 2006 12 1412 1419 10.1158/1078-0432.CCR-05-1906 16533763

[B21] Kim TY Jong HS Song SH Dimtchev A Jeong SJ Lee JW Kim TY Kim NK Jung M Bang YJ Transcriptional silencing of the DLC-1 tumor suppressor gene by epigenetic mechanism in gastric cancer cells Oncogene 2003 22 3943 3951 10.1038/sj.onc.1206573 12813468

[B22] Seng TJ Low JS Li H Cui Y Goh HK Wong ML Srivastava G Sidransky D Califano J Steenbergen RD Rha SY Tan J Hsieh WS Ambinder RF Lin X Chan AT Tao Q The major 8p22 tumor suppressor DLC1 is frequently silenced by methylation in both endemic and sporadic nasopharyngeal, esophageal, and cervical carcinomas, and inhibits tumor cell colony formation Oncogene 2007 26 934 944 10.1038/sj.onc.1209839 16862168

[B23] Goodison S Yuan J Sloan D Kim R Li C Popescu NC Urquidi V The RhoGAP protein DLC-1 functions as a metastasis suppressor in breast cancer cells Cancer Res 2005 65 6042 6053 10.1158/0008-5472.CAN-04-3043 16024604 PMC1360170

[B24] Yuan BZ Durkin ME Popescu NC Promoter hypermethylation of DLC-1, a candidate tumor suppressor gene, in several common human cancers Cancer Genet Cytogenet 2003 140 113 117 10.1016/S0165-4608(02)00674-X 12645648

[B25] Yuan BZ Zhou X Durkin ME Zimonjic DB Gumundsdottir K Eyfjord JE Thorgeirsson SS Popescu NC DLC-1 gene inhibits human breast cancer cell growth and in vivo tumorigenicity Oncogene 2003 22 445 450 10.1038/sj.onc.1206064 12545165

[B26] Healy KD Hodgson L Kim TY Shutes A Maddileti S Juliano RL Hahn KM Harden TK Bang YJ Der CJ DLC-1 suppresses non-small cell lung cancer growth and invasion by RhoGAP-dependent and independent mechanisms Mol Carcinog 2008 47 326 337 10.1002/mc.20389 17932950 PMC2679972

[B27] Ullmannova V Popescu NC Inhibition of cell proliferation, induction of apoptosis, reactivation of DLC1, and modulation of other gene expression by dietary flavone in breast cancer cell lines Cancer Detect Prev 2007 31 110 118 10.1016/j.cdp.2007.02.005 17418982 PMC1950447

[B28] Beier JI Arteel GE Alcoholic liver disease and the potential role of plasminogen activator inhibitor-1 and fibrin metabolism Exp Biol Med (Maywood) 2012 237 1 9 10.1258/ebm.2011.011255 22238286 PMC5047512

[B29] Rau JC Beaulieu LM Huntington JA Church FC Serpins in thrombosis, hemostasis and fibrinolysis J Thromb Haemost 2007 5 Suppl 1 102 115 17635716 10.1111/j.1538-7836.2007.02516.x PMC2670448

[B30] Malinowsky K Wolff C Berg D Schuster T Walch A Bronger H Mannsperger H Schmidt C Korf U Höfler H Becker KF uPA and PAI-1-Related Signaling Pathways Differ between Primary Breast Cancers and Lymph Node Metastases Transl Oncol 2012 5 98 104 22496926 10.1593/tlo.11268 PMC3323931

[B31] Dorn J Harbeck N Kates R Gkazepis A Scorilas A Soosaipillai A Diamandis E Kiechle M Schmalfeldt B Schmitt M Impact of expression differences of kallikrein-related peptidases and of uPA and PAI-1 between primary tumor and omentum metastasis in advanced ovarian cancer Ann Oncol 2011 22 877 883 10.1093/annonc/mdq462 20924077

[B32] Gupta A Lotan Y Ashfaq R Roehrborn CG Raj GV Aragaki CC Montorsi F Shariat SF Predictive value of the differential expression of the urokinase plasminogen activation axis in radical prostatectomy patients Eur Urol 2009 55 1124 1133 10.1016/j.eururo.2008.06.054 18585843

[B33] Hofmann R Lehmer A Buresch M Hartung R Ulm K Clinical relevance of urokinase plasminogen activator, its receptor, and its inhibitor in patients with renal cell carcinoma Cancer 1996 78 487 492 10.1002/(SICI)1097-0142(19960801)78:3<487::AID-CNCR16>3.0.CO;2-V 8697395

[B34] Hofmann R Lehmer A Hartung R Robrecht C Buresch M Grothe F Prognostic value of urokinase plasminogen activator and plasminogen activator inhibitor-1 in renal cell cancer J Urol 1996 155 858 862 10.1016/S0022-5347(01)66328-6 8583593

[B35] Papadopoulou S Scorilas A Yotis J Arnogianaki N Plataniotis G Agnanti N Talieri M Significance of urokinase-type plasminogen activator and plasminogen activator inhibitor-1 (PAI-1) expression in human colorectal carcinomas Tumour Biol 2002 23 170 178 10.1159/000064033 12218297

[B36] Cai Z Li YF Liu FY Feng YL Hou JH Zhao MQ Expression and clinical significance of uPA and PAI-1 in epithelial ovarian cancer Ai Zheng 2007 26 312 317 17355798

[B37] Koensgen D Mustea A Denkert C Sun PM Lichtenegger W Sehouli J Overexpression of the plasminogen activator inhibitor type-1 in epithelial ovarian cancer Anticancer Res 2006 26 1683 1689 16617562

